# Supplementary material for: Prevalence and associated risk factors of general and abdominal obesity in rural and urban women in Bangladesh
Source: PLoS One. 2020 May 29;15(5):e0233754. doi: 10.1371/journal.pone.0233754 (PMC7259624; doi:10.1371/journal.pone.0233754)
Supplement: Attachment — Submitted filename: Review Manuscript Number PONE.docx [file pone.0233754.s001.docx]

**Review Manuscript Number PONE-D-19-33594
"Prevalence and associated risk factors of general and abdominal obesity in rural and urban women in Bangladesh"**

This article addresses an important public health issue not just in Bangladesh but globally. With many LMIC populations undergoing epidemiological transitions, it is crucial to understand contextual differences in obesity levels in order to tailor interventions appropriately. The comparison between rural and urban is therefore a key addition to the body of knowledge.

The statistical analyses are technically sound and appropriate conclusions have been drawn from the data. However, the overall language and grammar of the manuscript needs a major review for clarity, if possible working with a copy-editor. The methods section in my view also needs further clarification.

**MAJOR REVISIONS**

1. I recommend that the authors work with a copy-editor in reviewing language and grammar of the entire manuscript. Because the paper not only compares rural and urban but also the different regions, the language needs to be clear and precise to get the right story across. The discussion section will also benefit immensely from language review in order to bring forward the true value of this study.

Examples of unclear areas: page 2 lines 19/20

"An increasing trend of abdominal obesity

19 was found among the participants with increasing age except for the Chattragram region,

20 {whereas}, the prevalence of general obesity was comparatively higher at middle age to 60 years

21 participants."

Also page 7, line 9; page 8 line 6-7.

2. Methods:

a) More detail can be added to the description of the setting eg How were the divisions selected? Out of a total of how many? This helps the reader to see generalisability of the study. What is the population size of these regions? Do they each have the same proportion of rural to urban area?

b) How was the sample size arrived at? The Sylhet region has a noticeably smaller sample size compared to the others - why was this? A discussion of potential implications may be necessary too.

c) Physical activity categories: it might be useful to show the classes of jobs or occupation that were used to arrive at the physical activity groups and whether this follows any other connvetion or tool used in quantifying physical activity. This is especially important because there is a sizeable proportion of housewives - how does this occupation the classification of physical activity.

**MINOR REVISIONS**

Results: It brings better clarity if the reference category is mentioned when reporting regression results, and to indicate the direction of the association, especially in the abstract.
