# Supplementary material for: Prevalence and associated risk factors of general and abdominal obesity in rural and urban women in Bangladesh
Source: PLoS One. 2020 May 29;15(5):e0233754. doi: 10.1371/journal.pone.0233754 (PMC7259624; doi:10.1371/journal.pone.0233754)
Supplement: Attachment — Submitted filename: Response to reviewer comments.docx [file pone.0233754.s003.docx]

Date: 05 May 2020

Prof. Benn Sartorius
Academic Editor, PLOS ONE

Manuscript ID: PONE-D-19-33594R1

Dear Editor,

We acknowledge and appreciate the editor and reviewers suggestions for further improving our manuscript. We have carefully considered all the valuable comments and made revisions accordingly. We have revised the entire manuscript text once again to avoid language and grammatical errors. Text changes are marked in colour in the attached revised manuscript. We feel that we satisfactorily responded to all the issues raised and are looking forward to your reconsideration of our manuscript.

With best regards,

Farjana Islam

Department of Biochemistry and Molecular Biology

Shahjalal University of Science and Technology, Sylhet, Bangladesh

E-mail: farjanaislam308@gmail.com

**List of changes made in response to the reviewers**

**Responses to Reviewer #2**

Reviewer #2: The manuscript is now methodologically sound and sufficient discussion has been given regards study weaknesses and further research. However I feel the grammar and language still need further revision to present these finding s in the clearest possible way. Below are more examples that may need to be revised:

Response: We appreciate the valuable comments and suggestions. According to the suggestion we have revised the manuscript text to avoid the language and grammatical errors.

• Page 4 line 1-8; line 16-18

Response: The text has been revised (page 4 line 1-9 and line 15-16)

• Page 5 line 6: should be “aged above 18”
Line 9-11

Response: The word has been corrected according to suggestion. Moreover, lines 9-11 have been revised to clear the meaning (page 5 line 9-11)

• Page 7 line 14-15

Response: The sentences have been revised (page 7 line 13-14).

• Page 8 line 12: should be “higher prevalence”

Response: Thanks for marking the error. This has now been corrected.

• Page 10 line 4-5: “ documented” not documents…”higher than that reported”…
Line 8: “than that reported”
Line 9-11: “the prevalence of general obesity was found to have increased almost 3 fold…”

Response: The words have been corrected according to the suggestion (Page 10 line 3-9)

• Page 11 lines 1-3
Line 16-17

Response: The sentences have been revised. (page 11 line 1-2 and line 13-16).

• Page 12 line: should be “a significant portion of the participants did not agree to participate in the study”

Response: The sentence has been edited based on suggestion.
